# Supplementary material for: Trends in Phenolic Profiles of Achillea millefolium from Different Geographical Gradients
Source: Plants (Basel). 2023 Feb 7;12(4):746. doi: 10.3390/plants12040746 (PMC9964219; doi:10.3390/plants12040746)
Supplement: Supplementary file 1 [file plants-12-00746-s001.zip › plants-2200566-supplementary.pdf]

Supplementary Materials

Table S1. Pearson correlation coefficients (r) and significance value (*p*) between the content of individual phenolic compounds in inflorescences, leaves and stems of *Achillea millefolium* and the latitude and longitude of population sites in Turkey and Lithuania

**Compounds**	**Latitude**						**Longitude**					
	**Inflorescences**		**Leaves**		**Stems**		**Inflorescences**		**Leaves**		**Stems**	
	**r**	***p***	**r**	***p***	**r**	***p***	**r**	***p***	**r**	***p***	**r**	***p***
Neochlorogenic acid	0.80	< 0.001	0.63	< 0.001	-0.09	0.593	-0.76	< 0.001	-0.57	< 0.001	0.11	0.512
Chlorogenic acid	0.87	< 0.001	0.82	< 0.001	0.83	< 0.001	-0.84	< 0.001	-0.79	< 0.001	-0.83	< 0.001
4-*O*-caffeoylquinic acid	-0.33	0.051	-0.70	< 0.001	0.25	0.140	0.33	0.052	0.68	< 0.001	-0.27	0.107
3,4-*O*-dicaffeoylquinic acid	0.47	0.004	0.77	< 0.001	0.69	< 0.001	-0.45	0.005	-0.76	< 0.001	-0.74	< 0.001
3,5-*O*-dicaffeoylquinic acid	0.90	< 0.001	0.78	< 0.001	0.66	< 0.001	-0.88	< 0.001	-0.74	< 0.001	-0.67	< 0.001
1,5-*O*-dicaffeoylquinic acid	0.86	< 0.001	0.79	< 0.001	0.76	< 0.001	-0.84	< 0.001	-0.77	< 0.001	-0.76	< 0.001
4,5-*O*-dicaffeoylquinic acid	0.83	< 0.001	0.81	< 0.001	0.68	< 0.001	-0.82	< 0.001	-0.81	< 0.001	-0.68	< 0.001
Caffeic acid	0.64	< 0.001	0.43	0.008	0.46	0.005	-0.60	< 0.001	-0.39	0.017	-0.46	0.005
Quercitrin	0.42	0.010	0.73	< 0.001	0.74	< 0.001	-0.38	0.022	-0.71	< 0.001	-0.71	< 0.001
Rutin	-0.07	0.681	0.27	0.108	0.23	0.187	0.09	0.582	-0.26	0.122	-0.24	0.161
Quercetin	-0.20	0.241	-0.50	0.002	-0.52	0.001	0.16	0.351	0.45	0.006	0.50	0.002
Isoquercitrin	-0.61	0.000	0.13	0.456	0.32	0.057	0.63	< 0.001	-0.16	0.351	-0.35	0.038
Luteolin	-0.48	0.003	-0.21	0.208	0.28	0.092	0.46	0.005	0.23	0.180	-0.28	0.098
Luteolin-7-*O*-glucoside	0.51	0.002	0.51	0.001	0.28	0.102	-0.45	0.006	-0.50	0.002	-0.32	0.061
Luteolin-7-*O*-rutinoside	-0.04	0.797	0.79	< 0.001	-0.09	0.585	0.09	0.612	-0.76	< 0.001	0.06	0.717
Luteolin-*O*-3,7-diglucoside	-0.69	< 0.001	-0.20	0.239	–	–	0.71	< 0.001	0.22	0.205	–	–
Apigenin	0.79	< 0.001	-0.14	0.407	–	–	-0.80	< 0.001	0.14	0.404	–	–
Apigenin-7-*O*-glucoside	0.93	< 0.001	0.70	< 0.001	0.77	< 0.001	-0.92	< 0.001	-0.68	< 0.001	-0.80	< 0.001
Santin	-0.69	< 0.001	-0.17	0.313	-0.41	0.013	0.71	< 0.001	0.12	0.484	0.41	0.014
Total	0.81	< 0.001	0.83	< 0.001	0.80	< 0.001	-0.78	< 0.001	-0.80	< 0.001	-0.81	< 0.001

Table S2. The mean quantities (µg/g, DM) of phenolic compounds in inflorescences, leaves and stems of *Achillea millefolium* populations from Nevşehir (1) and Gaziantep (2) provinces in Turkey and comparison of their differences (p ≤ 0.05) according to *t*-test.

**Compounds**	**Inflorescences**				**Leaves**				**Stems**			
	**M (1)**	**M (2)**	**t[1]**	***p***	**M (1)**	**M (2)**	**t**	***p***	**M (1)**	**M (2)**	**t**	***p***
Neochlorogenic acid	208.0	229.2	-1.12	0.283	486.0	612.9	-0.88	0.391	252.1	275.4	-0.65	0.528
Chlorogenic acid	1045.3	1753.8	-2.12	0.052	4372.8	3959.5	0.24	0.813	1281.8	902.7	1.25	0.230
4-O-caffeoylquinic acid	1419.5	1318.6	0.38	0.708	1678.6	1426.3	0.72	0.485	617.4	660.2	-0.35	0.734
3,4-*O*-dicaffeoylquinic acid	1648.9	1741.5	-0.18	0.861	1232.2	789.1	1.01	0.329	260.5	126.0	2.76	0.015
3,5-*O*-dicaffeoylquinic acid	1716.2	2948.6	-2.70	0.017	2912.1	4401.3	-1.42	0.179	875.2	662.2	1.38	0.188
1,5-*O*-dicaffeoylquinic acid	0.0	0.0	–	–	45.5	56.7	-0.94	0.363	58.9	68.7	-0.75	0.463
4,5-*O*-dicaffeoylquinic acid	327.9	311.0	0.26	0.800	113.3	165.8	-1.12	0.281	139.7	78.0	2.19	0.046
Caffeic acid	3.7	0.0	1.15	0.271	24.9	43.0	-1.57	0.139	20.1	10.1	1.12	0.283
Quercitrin	0.0	0.0	–	–	0.0	0.0	–	–	0.0	0.0	–	–
Rutin	148.7	219.4	-1.18	0.257	2196.1	1810.2	0.56	0.586	547.2	728.9	-0.98	0.346
Quercetin	45.7	25.2	1.65	0.121	35.9	25.5	1.52	0.151	33.1	29.0	0.90	0.381
Isoquercitrin	243.1	566.5	-1.55	0.143	101.2	19.5	1.24	0.235	0.0	0.0	–	–
Luteolin	3920.7	1750.0	2.38	0.032	194.5	551.3	-0.76	0.459	127.6	114.6	0.70	0.497
Luteolin-7-*O*-glucoside	1200.6	2223.7	-2.46	0.028	178.8	327.2	-0.59	0.562	77.5	26.2	2.21	0.044
Luteolin-7-*O*-rutinoside	404.2	754.8	-1.83	0.089	147.2	170.2	-0.34	0.742	240.4	194.3	0.72	0.483
Luteolin-*O*-3,7-diglucoside	1317.2	2240.2	-1.24	0.237	0.0	186.0	-0.88	0.396	0.0	0.0	–	–
Apigenin	224.6	61.6	1.93	0.075	14.7	22.7	-0.28	0.785	0.0	0.0	–	–
Apigenin-7-*O*-glucoside	208.2	268.0	-0.54	0.600	0.0	40.8	-0.88	0.396	2.5	0.0	1.15	0.271
Santin	376.5	600.3	-2.14	0.051	444.1	203.4	2.31	0.036	231.9	232.8	-0.12	0.906
Total	14459.1	17012.5	-1.18	0.258	14177.6	14811.3	-0.17	0.865	4765.8	4109.2	0.96	0.355

[1] df = 14
